# Collective Intergenerational Trauma in Children and Families: Transmission Mechanisms, Effects and Therapeutic Interventions by Health and Social Work Professionals to Promote Health and Strengthen Psychological Resilience

**DOI:** 10.3390/healthcare14152343

**Published:** 2026-08-01

**Authors:** Danai Sofia Pyliou, Areti Stavropoulou, Michael Rovithis, Georgios Filippidis, Maria Moudatsou

**Affiliations:** 1 Department of Social Work, School of Health Sciences, Hellenic Mediterranean University, 71410 Heraklion, Greece; moudatsoum@hmu.gr; 2 Department of Nursing, Faculty of Health Science, West Attica University, 12243 Athens, Greece; astavropoulou@uniwa.gr; 3 Department of Business Administration & Tourism, School of Management and Economics Sciences, Hellenic Mediterranean University, 71410 Heraklion, Greece; rovithis@hmu.gr; 4 Department of Social Work, Democritus University of Thrace, 69132 Komotini, Greece; geofilip@sw.duth.gr

**Keywords:** collective trauma, intergenerational trauma, trauma transmission, trauma-informed interventions, health professionals

## Abstract

Collective intergenerational trauma is a complex phenomenon affecting descendants and families exposed to the enduring effects of collective traumatic events, such as forced displacement, genocide and war. This systematic review aimed to explore the mechanisms through which collective trauma is transmitted across generations, examine its consequences for children and families, and critically analyse interventions employed by health professionals and social workers. A systematic review was conducted using ScienceDirect, Wiley Online Library and PubMed. Studies published between 2016 and 2026 were screened for eligibility, resulting in the inclusion of 22 studies. The indexing terms “collective intergenerational trauma”, “collective transgenerational trauma”, “trauma transmission”, “transmission mechanisms”, “consequences”, “intergenerational trauma interventions”, “Social Care”, and “Health Professionals”, were used, along with the Boolean operators AND and OR. The included studies were synthesised using thematic analysis. The findings indicate that collective intergenerational trauma is transmitted through interacting family, communicative, sociocultural and biological mechanisms. Its consequences include psychological and psychosocial difficulties among descendants, disruptions in family relationships and adverse effects on family functioning. Existing interventions primarily focus on individual trauma, with limited attention to its collective and intergenerational dimensions. The findings highlight the need for culturally responsive, trauma-informed and family-centred interventions addressing collective intergenerational trauma within a systemic framework. Health professionals and social workers can play an important role in promoting mental health, strengthening resilience, mitigating the long-term effects of trauma and supporting affected children, families and communities.

## 1. Introduction

Large-scale traumatic events, such as war, genocide and forced displacement, may have enduring consequences not only for those directly exposed but also for their families and subsequent generations. Contemporary research indicates that these consequences may influence descendants’ psychological development, interpersonal relationships and family functioning [1,2]. The persistence of such effects across generations has contributed to increasing scholarly interest in collective intergenerational trauma and the processes through which collective traumatic experiences continue to affect descendants.

Collective intergenerational trauma is a complex and multidimensional phenomenon involving the persistence of psychological, familial and social effects of collective traumatic experiences across generations. Its transmission may occur through interacting psychological, familial, communicative, sociocultural and potentially biological processes [3,4]. Collective experiences and social memory may shape the behaviours, representations and relationships of subsequent generations [5], while alterations in stress regulation and gene expression have been proposed as possible biological pathways associated with increased psychological and physical vulnerability [1,6,7].

Given the multidimensional nature of the phenomenon and the diversity of perspectives through which it has been examined, conceptual distinctions between related forms of trauma are necessary. Although these concepts are interrelated, they are not synonymous. Collective intergenerational trauma refers specifically to the enduring consequences of large-scale traumatic experiences affecting a group and its descendants [8]. Historical trauma emphasises the cumulative effects of traumatic events experienced by a group sharing a common cultural, ethnic or social identity [9]. Intergenerational trauma refers more broadly to the processes through which the effects of trauma in one generation influence subsequent generations that did not directly experience the original event [2]. Secondary traumatic stress is conceptually distinct, as it concerns trauma-related symptoms resulting from indirect exposure to another person’s traumatic experiences [10].

Beyond these conceptual distinctions, understanding how the effects of collective trauma persist across generations requires consideration of the multiple processes involved in their transmission. The transmission of collective intergenerational trauma cannot be explained by a single pathway. Family and psychosocial factors appear to play a central role. Parenting practices, communication patterns and parental disclosure or silence regarding traumatic experiences may influence how trauma-related effects are experienced by children [11]. Parental behaviour and the wider family environment may also reinforce these effects [3]. Psychodynamic approaches further emphasise the influence of unresolved traumatic experiences and unconscious emotional processes on parent–child relationships and descendants’ psychological development [12]. The interaction of these transmission processes may contribute to a range of psychological, interpersonal, social and physical consequences among descendants.

Intergenerational trauma has been associated with psychological and psychosocial difficulties among descendants who were not directly exposed to the original event [13,14]. Reported consequences include anxiety and depression [15,16] and post-traumatic stress symptoms [17]. Fear and mistrust may impair social integration [18], contribute to social withdrawal [19] and hinder the development of trusting interpersonal relationships [20].

The multidimensional nature of these consequences highlights the need for interventions that extend beyond the treatment of individual trauma symptoms. Health professionals and social workers may play an important role in recognising intergenerational patterns, supporting children and caregivers, strengthening resilience, and implementing culturally responsive, trauma-informed and family-centred interventions. However, the development of such comprehensive responses is complicated by important conceptual, theoretical and empirical limitations in the existing literature.

Despite increasing research interest, existing studies approach collective intergenerational trauma from diverse theoretical and methodological perspectives, emphasising different transmission mechanisms and consequences. This heterogeneity, together with the tendency to examine transmission mechanisms, effects on children and families, and intervention approaches separately, limits an integrated understanding of how collective traumatic experiences persist within family and social systems. Addressing this fragmentation requires a synthesis of the available evidence that considers these dimensions together rather than as separate areas of investigation.

Accordingly, this systematic review examines the mechanisms through which the consequences of collective trauma may be transmitted across generations, their effects on children and family functioning, and the interventions employed by health professionals and social workers. By integrating these dimensions within a collective, child-focused and family-centred framework, the review aims to provide a more comprehensive understanding of collective intergenerational trauma and to support the development of systemic approaches to its prevention and management.

## 2. Aim and Research Questions

The research questions and eligibility criteria were primarily structured according to the Population–Concept–Context (PCC) framework, as it was considered appropriate for the focus of the review on collective intergenerational trauma and the inclusion of diverse study designs and methodological approaches. For the research question concerning evaluated interventions, the Population–Intervention–Comparison–Outcome (PICO) framework was additionally applied, where applicable, depending on the design and characteristics of the included intervention studies.

Accordingly, the systematic review addressed the following research questions:

1. Through which mechanisms is collective intergenerational trauma transmitted to children and families affected by collective traumatic experiences?
2. What are the consequences of collective intergenerational trauma for children and the family system in the context of collective traumatic experiences?
3. What interventions have been empirically evaluated and are used by professionals, including social workers, to address collective intergenerational trauma or its trauma-related consequences among survivors, descendants, children, and families affected by collective traumatic experiences?

## 3. Materials and Methods

### 3.1. Design

A systematic review involves a structured and rigorous approach to locating and evaluating literature relevant to a clearly defined topic. Using predefined criteria, the available evidence is systematically examined, integrated, and reported in a comprehensive manner in order to provide answers to the established research questions. Consequently, this research employed a systematic methodological framework for its examination that was conducted in April 2026 using the ScienceDirect, Wiley Online Library and PubMed databases. The main search terms included “collective intergenerational trauma”, “collective transgenerational trauma”, “trauma transmission”, “transmission mechanisms”, “consequences”, “intergenerational trauma interventions”, “Social Care”, and “Health Professionals”, along with the Boolean operators AND and OR, as shown in Table 1. Free-text keywords were used because equivalent controlled vocabulary was not consistently available across all databases. Filters were applied to restrict the search to English-language publications published between 2016 and 2026. Where available, the full-text filter was also applied.

### 3.2. Identifying Relevant Studies and Study Selection

A systematic literature search was conducted, and the selection of studies followed the main stages of the PRISMA framework. This process was presented using the PRISMA 2020 (Preferred Reporting Items for Systematic Reviews and Meta-Analyses) flow diagram, which was adopted as a framework for the transparent and systematic reporting of the literature search and study selection process [21]. In this way, all stages of the literature search, the reasons for study exclusion, and the final composition of the systematic review sample were clearly documented [22].

**Figure 1:**
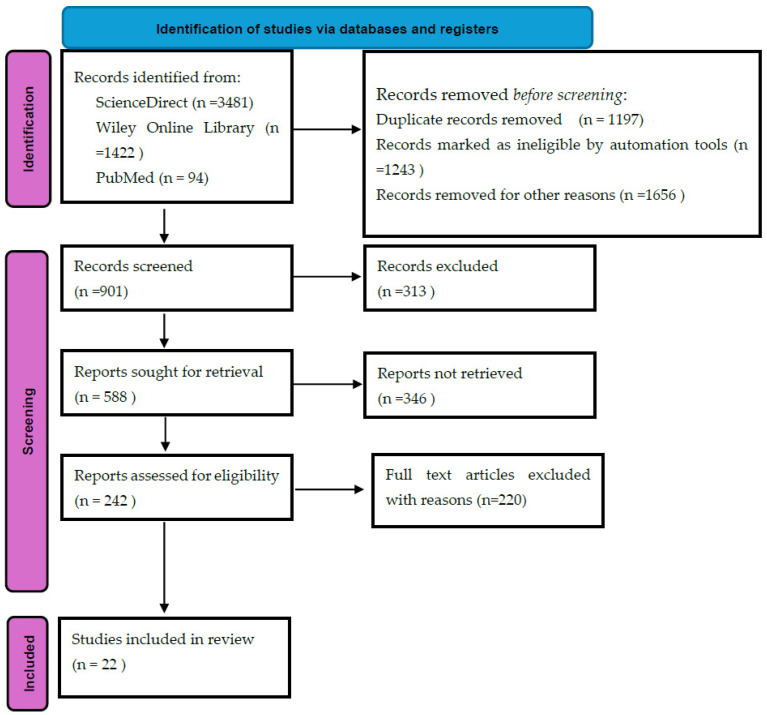
Flowchart illustrating the process of search and selection of studies (PRISMA).

Figure 1 illustrates the study selection process in accordance with the PRISMA 2020 guidelines. Searches of the ScienceDirect, Wiley Online Library, and PubMed databases identified 4.997 records. After duplicate removal and preliminary screening, records meeting the screening criteria proceeded to full-text assessment. Following the application of the predefined eligibility criteria, 22 studies were included in the final systematic review.

The study selection process was guided by predefined inclusion criteria. With regard to study design, quantitative, qualitative, and mixed-methods studies, as well as case studies and reflective papers, were included if they examined collective intergenerational trauma, its transmission mechanisms, its consequences for children and families, or interventions aimed at preventing or managing its effects. To ensure the timeliness of the review and its relevance to contemporary research on collective intergenerational trauma, the literature search was restricted to studies published between 2016 and 2026. Regarding publication type, only articles published in peer-reviewed scientific journals were included [22]. In addition, only studies published in English and available in full text were considered eligible for inclusion.

Essays, editorials, book reviews, and policy reports that did not provide sufficiently documented evidence relevant to the research questions were excluded. Studies lacking a clearly defined research design or an adequately described process of data collection and analysis were also excluded [23].

All records identified through the literature search were imported into Zotero 7 (Corporation for Digital Scholarship, Vienna, VA, USA) for reference management and duplicate detection. Duplicate records retrieved from different electronic sources were identified and removed before the screening process. Following duplicate removal, the titles and abstracts of the remaining records were screened to assess their relevance to the objectives and research questions of the review and their consistency with the predefined eligibility criteria. Records that were clearly unrelated to the scope of the review were excluded at this stage.

Following title and abstract screening, potentially relevant studies proceeded to full-text review. The eligibility of each article was independently evaluated against the predefined inclusion and exclusion criteria before a final decision regarding its inclusion in the review was made. Studies that did not meet the eligibility criteria were excluded, and the specific reasons for exclusion at the full-text assessment stage were systematically recorded. The studies that fulfilled all eligibility criteria constituted the final sample included in the systematic review.

The complete study selection process is presented in Figure 1.

### 3.3. Data Extraction

Following the completion of the study selection process, data from the included studies were systematically extracted and organized in Microsoft Excel 2021 (Microsoft Corporation, Redmond, WA, USA) spreadsheets. This procedure supported the structured management, comparison, and subsequent synthesis of the available evidence.

Data extraction was conducted in a consistent and standardized manner to ensure the comparable recording of the principal characteristics and findings of each included study [22]. Specifically, information was recorded regarding the author or authors, year of publication, country in which the study was conducted, study objectives, study population, sample size, research design, and key findings.

The principal findings of each study were also extracted according to their relevance to the research questions of the review, including evidence concerning the mechanisms of transmission of collective intergenerational trauma, its consequences for children and the family system, and the interventions developed to address collective intergenerational trauma or reduce its trauma-related effects.

### 3.4. Data Synthesis

Given the considerable heterogeneity of the included studies in terms of research design, participant characteristics, data collection and measurement instruments, and analytical approaches, a narrative and thematic synthesis was adopted rather than a statistical meta-analysis. This approach was considered appropriate because it enables the integration and interpretation of heterogeneous qualitative and quantitative evidence and is particularly suitable for systematic reviews in the social sciences [24].

The findings of the included studies were compared and grouped according to conceptual similarities and their relevance to the research questions of the review. Through this process, the evidence was organized into broader, conceptually coherent thematic categories. Studies addressing more than one research question contributed findings to more than one thematic category; however, only the findings directly relevant to each research question were synthesized within the corresponding section.

The synthesis was organized into three principal thematic areas: (1) mechanisms of transmission of collective intergenerational trauma, (2) consequences of collective intergenerational trauma for children and the family system, and (3) empirically evaluated interventions addressing collective intergenerational trauma or its trauma-related consequences. Within each thematic area, findings from studies employing different methodological approaches were narratively integrated in order to identify recurring patterns, convergent and divergent findings, and gaps in the available evidence.

For transparency and to provide readers with a comprehensive overview of the evidence included in this review, the characteristics of the 22 selected studies are presented in Appendix A (Table A1).

### 3.5. Quality Assessment

No formal quantitative appraisal of methodological quality or risk of bias was conducted using standardized assessment tools, such as the Cochrane Risk of Bias tools, GRADE, CASP, JBI, or MMAT. This decision reflected the aims and scope of the review, as well as the substantial methodological diversity of the included evidence. The review incorporated qualitative, quantitative, and mixed-methods studies that differed considerably in their research designs, data collection methods, and analytical approaches. Given this heterogeneity, the application of a single standardized appraisal instrument was considered unsuitable, as it might not adequately capture the methodological differences among the included studies and could lead to an oversimplified assessment of the available evidence.

Nevertheless, methodological considerations were incorporated throughout the processes of study selection, inclusion, and evidence synthesis through a narrative qualitative appraisal of the methodological adequacy of the included studies. Each included study was assessed independently by two reviewers to reduce the potential for reviewer-related bias. Any disagreements arising during the appraisal process were discussed and resolved through consensus. This appraisal considered key methodological aspects, including the clarity of the research objectives, the appropriateness of the study design in relation to the stated aims, and the adequacy of the description of the study sample and research context. Additional consideration was given to the appropriateness of participant selection and the transparency and adequacy of the reported data collection procedures. The methodological appraisal therefore focused on whether the methods employed in each study were sufficiently described and appropriate for addressing the respective research objectives.

The methodological strengths and limitations identified through this qualitative appraisal were taken into consideration during the synthesis and interpretation of the evidence. This process supported a more critical evaluation of the findings and allowed for the conclusions of the review to be formulated in light of the methodological characteristics and limitations of the available evidence.

The absence of a formal standardized assessment of methodological quality and risk of bias is acknowledged as a limitation of the present review and was taken into account when interpreting the findings and drawing conclusions. However, the use of a narrative and interpretive approach to methodological appraisal was considered consistent with the broad and heterogeneous nature of the evidence synthesized in this review and with approaches applied in social science reviews that primarily aim to integrate and interpret diverse bodies of evidence rather than statistically compare intervention effects or establish causal relationships [22,23].

## 4. Results

### 4.1. Characteristics of the Included Studies

The studies included in this systematic review were conducted across diverse geographical regions, reflecting the international scope of the available literature. Table 2 summarizes the main characteristics of the included studies and their geographical distribution. Asia accounted for the largest proportion of studies (31.8%), followed by North America (22.7%). Europe and Oceania each contributed 13.6% of the included studies, while Africa and multinational studies each represented 9.1%. The publication years of the studies included in the review ranged from 2016 to 2026. More specifically, five studies (22.7%) were published between 2016 and 2018, three studies (13.6%) between 2019 and 2021, whereas the majority (n = 14, 63.6%) were published between 2022 and 2026.

Of the 22 studies included in the review, quantitative designs were the most frequently employed (n = 11), followed by qualitative designs (n = 8) and mixed-methods designs (n = 2). One publication was classified as a case study (n = 1).

The narrative synthesis of the studies included in this systematic review revealed a broad range of evidence concerning collective intergenerational trauma. To facilitate a coherent presentation of the findings, the synthesized evidence was organized into three thematic categories according to whether it related to (1) mechanisms through which collective intergenerational trauma affects children and families (n = 11 studies), (2) consequences of collective intergenerational trauma for children and the family system (n = 11 studies), or (3) interventions addressing collective intergenerational trauma and its consequences (n = 6 studies). Some studies contributed findings to more than one thematic category and were therefore included in the narrative synthesis of more than one research question; consequently, the number of studies across the three thematic categories exceeds the total number of studies included in the review.

### 4.2. Thematic Categories

#### 4.2.1. Μechanisms of Transmission of Collective Intergenerational Trauma

The mechanisms involved in the transmission of collective intergenerational trauma that affect children and families are associated with multiple factors. Specifically, Bonumwezi et al. [25], who conducted a study in Rwanda with a sample of 251 adult descendants of survivors of the Genocide against the Tutsi, investigated the familial and psychosocial factors involved in the intergenerational transmission of trauma. More specifically, the authors examined parents’ exposure to traumatic events and their symptoms of post-traumatic stress, parenting practices, and parent–child communication, as well as symptoms of post-traumatic stress, anxiety, and depression among the descendants. The results identified the communication of parental trauma as a key mechanism of intergenerational transmission, particularly through non-verbal communication and forms of communication that induce feelings of guilt among the descendants, as well as dysfunctional family communication patterns and negative parenting practices, such as indifference towards children and abusive parenting.

Johns et al. [26], in a study of 98 adult second- and third-generation descendants of Holocaust survivors, focused on the role of family communication. Frequent and direct communication about the historical event served as a central mechanism of trauma transmission, enhancing awareness of historical loss. Conversely, indirect forms of communication and guilt-inducing communication emerged as dysfunctional mechanisms of transmission, negatively affecting the mental well-being of the descendants.

Another study conducted in Australia with fourteen grandchildren of Holocaust survivors aimed to investigate the mechanisms of intergenerational transmission of intergenerational trauma. Family communication about the traumatic past, the preservation of historical memory and the meanings attributed to the Holocaust, as well as the emotional reactions of the ancestors to the trauma, combined with the participants’ relationships with other descendants of survivors, constituted key mechanisms of intergenerational transmission of trauma. Through these mechanisms, the trauma continued to affect the descendants, shaping the way they perceived and made sense of both their family history and their contemporary lives [27].

Lin et al. [28] conducted a study in the United States with the participation of 13 minors of Cambodian descent. The study aimed to investigate how younger generations learn about their families’ traumatic experiences, focusing on intergenerational communication, sources of information, learning opportunities, and the emotional factors that influence this process. The results showed that intergenerational communication constituted a key mechanism for transmitting the traumatic past, not only through parents and grandparents but also through community narratives and the media. At the same time, emotional closeness, motivation to learn, and tolerance of emotional discomfort facilitated direct and coherent communication, while the absence of these factors was associated with silence and emotionally charged communication.

Isobel et al. [29] explored psychiatrists’ perceptions and clinical experiences regarding intergenerational trauma in a qualitative study involving 13 psychiatrists. The results of the study revealed that the intergenerational transmission of trauma occurs through parental psychological distress, reduced parenting capacity, disruption of attachment patterns, and the reproduction of trauma cycles within the family. Specifically, the reproduction of trauma cycles refers to the maintenance and repetition of dysfunctional patterns of relationships, behaviors, and emotional reactions associated with the original trauma and passed down from one generation to the next. In this way, the consequences of collective trauma affect family functioning and the relationships between family members, contributing to the maintenance and intergenerational transmission of trauma.

Kevers et al. [30] investigated the ways in which collective intergenerational trauma is transmitted within the family context in a qualitative study involving 10 Kurdish refugee parents and 17 children. The findings showed that the transmission of trauma occurs mainly indirectly, through silence and discussions about contemporary events of violence, as well as through collective historical memory, family narratives, and cultural symbols or objects that retain symbolic value in relation to the past.

The study, which was conducted with 422 adult children of Holocaust survivors, investigated what is transmitted from survivors to their descendants and through which familial and post-traumatic mechanisms intergenerational transmission occurs. The findings revealed that the transmission of trauma primarily occurs through the post-traumatic adjustment patterns developed by parents in response to their traumatic experiences. These patterns, such as the adoption of a victimization stance and emotional detachment, were shaped both by the survivors’ Holocaust experiences and family histories and by the conditions of the post-war family environment, and they influenced their relationships with their children. A particularly significant mechanism of intergenerational transmission was the disruption of emotional bonds between parents and children, which was linked, both directly and indirectly, to negative effects on the descendants. Conversely, living in Israel emerged as a protective sociocultural factor that could mitigate the negative effects of intergenerational transmission [31].

Shrira [32] conducted a study involving 450 participants that investigated the relationship between parental communication about the Holocaust and secondary traumatization as potential mechanisms of intergenerational transmission of trauma among the descendants of survivors. The results showed that intrusive parental communication about the Holocaust was associated with increased levels of secondary traumatization among the offspring. Secondary traumatization, in turn, was associated with more negative perceptions of aging and increased anxiety about aging and death. These findings highlight intrusive parental communication and secondary traumatization as interconnected mechanisms through which the psychological consequences of collective trauma can be transmitted to offspring and affect their mental health and perceptions of aging and death.

Krauskopf et al. [33] conducted a study involving 24 siblings from 11 families and investigated how adult children of Holocaust survivors experience and actively engage with their traumatic family history. The results revealed that the intergenerational transmission of collective trauma occurred through both explicit and implicit forms of communication. The implicit forms of transmission included silence, the transmission of fragmented information, and the indirect reception and internalization of the emotional burden of parental trauma by the descendants, even when it was not directly expressed in words. At the same time, family narratives, the active search for information about the family past, and the preservation of historical memory constituted mechanisms through which descendants actively connected with the family’s traumatic past, contributing to the intergenerational transmission and preservation of the traumatic legacy.

In a study conducted with 14 Armenian American descendants from seven families, the intergenerational impact of the Armenian Genocide on the cultural identity and lived experiences of the descendants was investigated. The results highlighted family narratives and the transmission of knowledge and memory about the historical trauma as key mechanisms of intergenerational transmission. At the same time, the transmission of emotional reactions, attitudes, and values through family interactions and daily practices contributed to the intergenerational transmission of trauma and its effects [34].

Peltonen et al. [35] investigated the familial mechanisms involved in the intergenerational transmission of collective trauma among 212 children and 94 mothers from Syrian refugee families, focusing on maternal post-traumatic stress and parental disciplinary practices. The findings highlighted maternal psychopathology as a key mechanism of trauma transmission through its impact on emotional responsiveness, emotional availability, and emotion regulation within the mother–child relationship. At the same time, the role of disciplinary practices was examined; however, these practices were not found to moderate the relationship between maternal post-traumatic stress and children’s emotional processing.

#### 4.2.2. Consequences of Collective Intergenerational Trauma for Children and the Family System

The review of the literature revealed multiple psychological, emotional, interpersonal, and psychosocial consequences of collective intergenerational trauma among descendants of populations exposed to collective traumatic events. Bonumwezi et al.’s [25] study was conducted with 251 adult descendants of survivors of the genocide against the Tutsi in Rwanda and investigated the familial and psychosocial mechanisms involved in the intergenerational transmission of trauma. The results showed that mothers’ PTSD symptoms were associated with PTSD, anxiety, and depression symptoms among the offspring. Additionally, fathers’ PTSD symptoms were indirectly associated with anxiety and depression among the offspring through their parenting practices.

Another study, conducted in the United States with 98 adult second- and third-generation descendants of Holocaust survivors, aimed to investigate the relationship of Jewish identity and family communication about the Holocaust with awareness of historical loss and the mental well-being of the descendants. The results of the data analysis showed that dysfunctional family communication regarding the Holocaust was associated with lower levels of mental well-being among the descendants. At the same time, more frequent family communication about the Holocaust and a stronger Jewish identity were associated with greater awareness of historical loss [26].

The study by Greenfeld et al. [27], conducted in Australia with 14 grandchildren of Holocaust survivors, explored the lived experiences of the descendants, the intergenerational transmission of trauma, and the impact of their ancestors’ traumatic experiences on subsequent generations. The findings showed that the consequences of collective intergenerational trauma remain evident even in later generations. Specifically, the descendants exhibited intense emotional reactions linked to the traumatic experiences of their ancestors, while inherited trauma influenced the way they perceived and interpreted their family history. At the same time, the descendants attributed particular significance to the Holocaust and their family’s traumatic legacy, highlighting the ongoing impact of collective trauma on their emotional experiences and on the way they related to their family past.

The study by Krauskopf et al. [33], conducted in Australia with 24 adult children of Holocaust survivors from 11 families, explored how descendants experience and actively engage with their traumatic family history. The findings highlighted both negative and positive consequences of the intergenerational transmission of trauma. Specifically, the descendants’ engagement with their parents’ traumatic experiences was associated with ambivalence and emotional distress, highlighting the ongoing psychological burden caused by the traumatic family legacy. At the same time, positive consequences were identified, such as recognition of parental resilience, meaning-making in relation to family history, the preservation and transmission of Holocaust testimony, and the provision of psychosocial support to their elderly parents who were Holocaust survivors.

The study by Lipari [34], conducted in the United States with 14 Armenian American descendants of Armenian Genocide survivors from seven families, explored the intergenerational impact of the Genocide on the cultural identity and lived experiences of the descendants. The findings revealed that the traumatic legacy of the Genocide was associated with secondary traumatization among the descendants. However, alongside the negative consequences, positive effects also emerged, such as the development of resilience, cultural pride, the strengthening of cultural identity, and the cultivation of cultural empathy. Moreover, the traumatic legacy significantly influenced the formation of the descendants’ cultural identity and strengthened their desire to preserve and transmit Armenian cultural heritage to future generations, thereby contributing to the preservation of the collective memory of the Genocide.

The study by Kaur and Jagg [36], conducted in India with 62 second- and third-generation descendants of survivors of the India–Pakistan Partition, investigated the consequences of collective intergenerational trauma for the children and grandchildren of the survivors. The findings revealed moderate levels of intergenerational trauma in both the second and third generations, demonstrating the long-term persistence of the impact of collective trauma on descendants. The absence of statistically significant differences between the second and third generations in the intensity of trauma suggests that its impact remains relatively stable and does not significantly diminish across generations. Therefore, the main consequence identified was the persistence of collective trauma over time and its continued psychological impact on descendants. At the same time, no significant differences were found between the two generations in their adaptation to traumatic experiences or in the positive changes that emerged following trauma.

Another study, conducted in Turkey with 100 Syrian refugee families, investigated the relationship between parental post-traumatic stress and children’s emotional processing abilities. The findings showed that higher levels of maternal post-traumatic stress were associated with reduced emotion recognition ability among children, which may have negative effects on their social interaction, empathy, and emotion regulation. In contrast, no corresponding relationship was found between paternal post-traumatic stress and children’s emotion recognition ability [37].

The study by Huynh et al. [38], conducted in the United States with 10 second-generation Southeast Asian American adults, explored experiences of collective intergenerational trauma and its effects on the descendants. The findings showed that the consequences of collective intergenerational trauma manifested as difficulties in identity formation, emotional detachment from the traumatic experiences of previous generations, feelings of guilt and responsibility toward the family, and intense cultural and intergenerational conflicts. At the same time, negative effects on mental health were identified, manifesting as anxiety, internalized psychological distress, and difficulties in expressing and regulating emotions. However, positive consequences also emerged, such as the development of a bicultural identity and the strengthening of family and community resilience.

The study by Bezo and Maggi [39], conducted in Ukraine with 45 participants from three successive generations, explored the intergenerational consequences of the collective trauma caused by the 1932–1933 Holodomor on the physical health and well-being of survivors and their descendants. The results showed that the consequences of collective trauma persisted across three generations and manifested as a higher prevalence of chronic health problems, such as cardiovascular disease, as well as anxiety and a reduced sense of well-being and security, negatively affecting the quality of life of subsequent generations.

The study by Bachem et al. [40], conducted in Israel with 127 Eritrean female asylum seekers with preschool-aged children, investigated the consequences of the intergenerational transmission of collective trauma by examining the relationships of maternal complex post-traumatic stress disorder (CPTSD), post-traumatic stress disorder (PTSD), and depression with children’s emotional and behavioral difficulties. The results showed that children of mothers with CPTSD exhibited increased internalizing and externalizing difficulties, which manifested as anxiety, social withdrawal, aggression, and difficulties in emotional regulation. Maternal CPTSD was more strongly associated with children’s difficulties than maternal PTSD, while the co-occurrence of CPTSD and depression was associated with increased internalizing problems, such as low self-esteem and emotional distress. The findings highlight the significant adverse impact on children’s emotional and behavioral development and the increased risk of future mental health difficulties.

Peltonen et al. [35] investigated the consequences of collective intergenerational trauma for children’s emotional development among 212 children and 94 mothers from Syrian refugee families, taking into account the broader context of displacement experiences and post-migration living conditions. The findings showed that maternal post-traumatic stress was associated with reduced emotional processing abilities among children, indicating difficulties in understanding, recognizing, and regulating their emotions.

#### 4.2.3. Interventions Addressing Collective Intergenerational Trauma or Its Trauma-Related Consequences

The review of the literature identified several empirically evaluated interventions aimed at addressing collective intergenerational trauma or its trauma-related psychological, psychosocial, and family consequences among survivors, descendants, children, and families affected by collective traumatic experiences. The study by Smaik et al. [41], conducted in Jordan with 40 adult Syrian refugees with PTSD, empirically evaluated Narrative Exposure Therapy (NET) as an intervention for addressing the psychological consequences associated with exposure to collective traumatic experiences. The intervention primarily aimed to reduce symptoms of PTSD, anxiety, and depression. The findings showed a significant reduction in these symptoms following the completion of the intervention, as well as high rates of treatment participation and adherence, treatment completion, and participant satisfaction. Therefore, NET emerged as a feasible, acceptable, and culturally appropriate intervention for addressing the psychological consequences of collective trauma among adult refugees.

The study by Ellis and Jones [42], conducted in 2022 in Cairo with seven Sudanese refugees with PTSD, empirically evaluated Narrative Exposure Therapy (NET) delivered by trained lay counselors in a community setting to address the psychological consequences associated with exposure to collective violence and forced displacement. After the intervention, a significant reduction in PTSD symptoms, anxiety, and overall psychological distress was recorded, while the reduction in depressive symptoms was not statistically significant. The findings highlighted the potential of delivering NET through trained lay counselors in community settings as an intervention to address the psychological consequences of collective traumatic experiences among refugee populations.

Kananian et al. [43] evaluated the effectiveness of culturally adapted cognitive behavioral therapy combined with problem management (CA-CBT+) among 24 male refugees in Germany, taking into account the effects of collective and intergenerational trauma that characterize refugee populations. The purpose of the study was to investigate whether this intervention could reduce psychological symptoms and enhance psychological resilience. The results showed significant improvements in general psychological distress, post-traumatic stress symptoms, depression, somatic symptoms, emotional regulation, and quality of life compared with the control group. Although the improvements partially diminished after one year, they remained significant compared with pre-intervention levels.

The study by Gill [44], conducted in nine communities across four continents with the participation of 150 young adults and approximately 500 individuals over the age of 50, explored the contribution of Intergenerational Dialogue and Inquiry (IDI) to breaking intergenerational silence, restoring cultural memory, and enhancing collective healing. The findings showed that the intervention strengthened cultural identity and dignity, intergenerational relationships, and understanding of systemic violence and discrimination, while also contributing to collective healing and community well-being.

The study by Neville et al. [45], conducted in New England, in the United States, with 80 refugee families, empirically evaluated the outcomes, feasibility, and acceptability of the Family Strengthening Intervention for Refugees (FSI-R), which aimed to address the psychosocial and familial consequences associated with refugee families’ exposure to collective traumatic experiences and forced displacement. The findings showed improvements in family communication, parental involvement, caregiver–child relationships, and supportive parenting practices. At the same time, Bhutanese children exhibited reductions in symptoms of depression, anxiety, and fear, as well as in behavioral problems. The intervention was generally deemed feasible and acceptable for addressing trauma-related consequences among refugee children and families, although its implementation was affected by linguistic, time-related, educational, and cultural factors.

The study by Jordans et al. [46], conducted in Lebanon with 240 female caregivers, empirically evaluated the effectiveness of the nine-session Caregiver Support Intervention (CSI) in enhancing psychosocial well-being and family functioning in contexts characterized by armed conflict. The findings showed that the intervention improved children’s psychosocial well-being in the short term through reductions in caregivers’ psychological distress, improvements in their well-being, and reductions in harsh parenting practices. The results highlight the potential of interventions focused on caregiver support and strengthening parenting functioning to reduce the family processes through which the consequences of collective trauma affect the next generation. However, the benefits of the intervention were not maintained at the three-month follow-up assessment.

## 5. Discussion

The present systematic review gathered and synthesized the findings of 22 primary studies from different countries and sociocultural contexts, with the aim of investigating the transmission mechanisms and consequences of collective intergenerational trauma, as well as the interventions that have been implemented to address trauma-related effects. Overall, the findings showed that collective trauma can affect subsequent generations through multiple and interacting mechanisms and can be associated with significant emotional, behavioral, interpersonal, and psychosocial consequences. These findings are consistent with previous studies, according to which collective traumatic events affect not only the mental health and well-being of directly exposed survivors but may also affect subsequent generations, even when they have not been directly exposed to the original traumatic event, and are transmitted intergenerationally through multiple mechanisms [3]. At the same time, interventions targeting survivors, descendants, children, and families appear to contribute to reducing the effects of trauma and enhancing psychosocial well-being and family functioning.

Regarding transmission mechanisms, the findings of the present review show that collective intergenerational trauma is transmitted through different but interconnected familial, communicative, emotional, and sociocultural mechanisms. Family communication emerged as one of the main transmission mechanisms. This finding is supported by the previous literature, which has shown that families of survivors of severe traumatic events often experience difficulties in family communication and emotional expression [47].

More specifically, nonverbal, indirect, guilt-inducing, and dysfunctional communication was associated with the transmission of trauma and negative psychological effects on descendants, as children are indirectly exposed to the psychological and emotional pain of their parents [25]. In contrast, direct and open communication appeared to help descendants better understand their family’s traumatic past [25,26]. At the same time, emotional closeness and the ability to manage emotional distress facilitated open communication, whereas their absence was associated with silence, fragmented narratives, and emotionally charged communication [28]. Similarly, previous studies have shown that silence, the withholding of information, and limited emotional expression can create a dysfunctional family environment, intensifying the emotional distress of subsequent generations [48]. Trauma also appeared to be transmitted indirectly through unspoken emotions, family narratives, symbolic objects, and the preservation of collective memory [30,33].

In addition to communication, parental psychological distress and post-traumatic symptoms played an important role in the transmission of trauma. These symptoms affected subsequent generations through reduced emotional availability, difficulties in parenting, negative parenting practices, and the repetition of dysfunctional family patterns [25,29,35]. This finding is consistent with other studies, according to which the trauma experienced by parents or caregivers may not only affect their parenting functioning but may also lead children to assume emotional and practical caregiving responsibilities [4]. This role reversal, described as parentification, can burden children’s mental health and hinder the development of their identity and autonomy [4,49].

Furthermore, the transmission of trauma was associated with disrupted attachment patterns and difficulties in the parent–child relationship [29]. Cerdeña et al. [3] reached a similar conclusion, arguing that parental dysregulation due to traumatic experiences may limit parents’ ability to provide stable and emotionally available care, contributing to the development of insecure attachment. Similarly, when parents carry their own early traumatic experiences, they may exhibit dysfunctional parenting behaviors and difficulties in responding to their children’s emotional needs [29,50]. At the same time, community narratives, cultural values, collective memory, and the meanings attributed to historical traumatic events also contributed to the preservation and transmission of trauma to subsequent generations [30,33].

Although biological transmission mechanisms did not emerge in the present review, other studies have investigated potential biological and epigenetic pathways [1,6]. According to the relevant literature, exposure to trauma may affect systems associated with the stress response and possibly gene expression, increasing vulnerability to psychological and physical difficulties [7].

Regarding the consequences of collective intergenerational trauma, parental post-traumatic stress was associated with symptoms of PTSD, anxiety, and depression in descendants, while negative parenting practices could further exacerbate psychological [25]. This finding was also reported in several other studies, which documented increased psychological burden in subsequent generations, mainly in the form of anxiety, depression, and PTSD symptoms [15,16,17,20]. Similar findings were reported specifically among adolescents who grew up with parents who had experienced traumatic events, as they exhibited increased symptoms of post-traumatic stress [14]. In addition, other studies showed that the psychological consequences in subsequent generations may extend beyond anxiety, depression, and PTSD and may include suicidal ideation, psychotic disorders, and broader psychosocial burden [18]. Similarly, Flanagan et al. [51] reported, in addition to the above symptoms, attention-deficit difficulties and severe psychosocial stress.

Children of parents who had experienced traumatic events also exhibited difficulties in recognizing, understanding, and regulating emotions, which may affect their emotional development and social relationships [35]. The studies by Cerdeña et al. [3] and Flanagan et al. [51] reached the same conclusion, linking collective intergenerational trauma to difficulties in emotional development, while emotional distancing was also reported as a possible consequence [13]. In addition, other studies showed that post-traumatic stress associated with collective traumatic experiences, particularly when it affects mothers, may negatively affect children’s ability to recognize, process, and express their emotions, leading to emotional difficulties and internal conflicts [37]. Some research findings also indicated that descendants may have an increased need for child psychiatric support, which has been associated with their exposure to their parents’ post-traumatic burden [52]. This need has been identified both in earlier research, such as Kellerman’s [53] study on the children of Holocaust survivors, and in more recent studies, which highlighted similar effects [52].

Children also exhibited anxiety, aggression, low self-esteem, emotional distress, and social withdrawal [40]. According to the findings of other studies, social isolation may indeed be a consequence of intergenerational trauma and may be associated with feelings of fear, distrust, and insecurity, which hinder social integration and increase wariness toward others [18]. This may lead to social withdrawal [17,19], difficulties in forming trusting relationships, and distancing from close interpersonal relationships [20].

Among adult descendants, collective intergenerational trauma was associated with lower mental well-being, emotional distress, secondary traumatization, guilt, a sense of responsibility toward the family, difficulties in identity formation, and cultural and intergenerational conflicts [26,27,33,34]. Several other studies focused on emotional distress as a consequence of collective intergenerational trauma, pointing out that descendants may struggle to invest time and psychological resources in their personal development and in the formation of meaningful interpersonal relationships [17]. Other studies referred more specifically to the feelings of guilt experienced by descendants in relation to the experiences and losses of previous generations [4,54].

The findings also showed that the consequences of collective trauma may remain evident across more than one generation. Similar levels of intergenerational trauma were identified among second- and third-generation descendants, suggesting that the psychological effects may persist over time [36]. Long-term consequences were also observed in physical health and overall well-being, such as chronic health problems, anxiety, reduced quality of life, and a lower sense of security [39]. In addition, this finding was also reported in other studies, which found that descendants of those exposed to collective trauma have an increased risk of developing physical health problems, such as cardiovascular diseases, diabetes, and obesity [55].

However, the effects of collective intergenerational trauma were not exclusively negative. Some descendants developed resilience, cultural pride, a stronger cultural or bicultural identity, and a greater ability to make meaning of their family history [34,36,38]. The traumatic family legacy also strengthened, among some descendants, the desire to preserve historical memory, cultural heritage, and family narratives, as well as to strengthen family and community bonds.

From the above, it can be concluded that a substantial part of the literature supports the association between previous generations’ exposure to traumatic events and the occurrence of psychopathological symptoms and other psychosocial or physical consequences in descendants [56,57]. On the other hand, there are also studies that question this association, as they did not identify clear adverse consequences among survivors’ descendants [58], while others did not find a significant association between intergenerational trauma and the mental health of the next generation [59]. These differences suggest that the consequences of collective intergenerational trauma do not manifest in the same way among all descendants but may be influenced by familial, sociocultural, and protective factors, as well as by methodological differences between studies.

Regarding interventions for addressing collective intergenerational trauma and its related consequences, the findings of the present review identified different therapeutic, family, and community approaches. Interventions that focused directly on processing traumatic experiences, such as Narrative Exposure Therapy (NET) and culturally adapted cognitive behavioral therapy (CA-CBT+), contributed to reducing symptoms of post-traumatic stress, anxiety, depression, and psychological distress, as well as to improving emotional regulation and the quality of life of refugees [41,42,43].

Similar findings were also reported in other studies, which identified NET as a promising approach for addressing complex traumatic symptoms and alleviating the consequences of PTSD, while also supporting its contribution to strengthening psychological resilience [41,60,61]. Similarly, other studies showed that CA-CBT may constitute an effective therapeutic approach, particularly when adapted to the cultural needs of the populations in which it is applied [62,63]. On the other hand, according to other studies, the overall effectiveness of this specific intervention continues to be evaluated, as the heterogeneity of the available research data does not yet allow for reliable and generalizable conclusions [64].

At the same time, family and community interventions contributed to improving family communication, parenting functioning, relationships between caregivers and children, and psychosocial well-being. In addition, they strengthened intergenerational relationships, cultural identity, and the preservation of collective memory [44,45,46]. These findings show that addressing the consequences of trauma is not limited to the individual level but may require interventions that take the family and community into account.

Overall, the findings show that interventions can reduce the consequences of trauma both in individuals and in families and communities. However, there are still few interventions that have been specifically designed for collective intergenerational trauma. A better understanding of the relationship between the mental health of parents and children may help in the development of more numerous and more appropriate interventions [65]. To achieve this, the multidisciplinary team plays a crucial role in promoting the health and psychological resilience of individuals. The multidimensional nature of collective intergenerational trauma makes it imperative to adopt coordinated interprofessional approaches, which go beyond piecemeal professional practices and fragmented models of care in health service delivery [66]. The cooperation of professionals of different specialties, such as social workers, nurses, psychologists and pediatricians, is considered necessary for the design and implementation of comprehensive and holistic care, which takes into account the needs, expectations, and specific characteristics of both the individual and the family [67].

The collaboration between professionals of different disciplines and the integration of diverse professional perspectives facilitate the early detection of the needs of the individual and the family, as well as the provision of safe and appropriate care services. At the same time, they contribute to the formation of a supportive framework that enhances the active participation and empowerment of both the individual and the family [66,68]. In addition, such collaborative practices have been associated with improved therapeutic outcomes, strengthening family resilience, preventing the intergenerational transmission of trauma, and providing comprehensive and high-quality health services [67].

### Limitations and Strengths

This systematic review provides a synthesis of the available evidence on collective intergenerational trauma, with particular emphasis on its transmission mechanisms, consequences for children and families, and interventions reported in the literature. A major strength of the review is the inclusion of studies employing diverse methodological approaches and examining different populations and collective trauma contexts, allowing for a broad and multidimensional understanding of this complex phenomenon. Furthermore, the integration of psychological, familial, sociocultural and biological perspectives, together with evidence on intervention approaches, provides a more holistic overview of the available evidence.

Despite the systematic search and selection process, several limitations should be acknowledged. First, only studies published in English between 2016 and 2026 were included, which may have resulted in the exclusion of relevant evidence published in other languages or outside the selected time frame. Moreover, the search was limited to four electronic databases, and relevant studies indexed exclusively in other databases may therefore not have been identified. Considerable heterogeneity was also observed among the included studies regarding study populations, research designs, methodological approaches, collective trauma contexts and intervention strategies. This heterogeneity limited direct comparisons across studies and the extent to which the findings can be applied across different populations and contexts.

## 6. Conclusions

The findings of this systematic review indicate that collective intergenerational trauma is a complex phenomenon transmitted through familial, communicative and sociocultural mechanisms, affecting descendants’ psychological wellbeing, interpersonal relationships and family functioning. There is still a lack of interventions specifically designed to address collective intergenerational trauma. Existing approaches mainly focus on individual symptoms, caregiver wellbeing and family functioning, without fully addressing the collective and intergenerational dimensions of trauma. Health professionals and social workers can play an important role in supporting affected children, families and communities and strengthening their resilience. Future research should focus on developing and evaluating interventions that directly address the collective and intergenerational nature of trauma and provide stronger evidence to guide effective professional practice.

## Figures and Tables

**Table 1:** Index using Boolean operators and specific terms.

Term	Boolean Operator	Term	Boolean Operator	Term
Collective Intergenerational Trauma	OR	Collective Transgenerational Trauma	AND	Trauma Transmission
Collective Intergenerational Trauma	OR	Collective Transgenerational Trauma	AND	Transmission Mechanisms
Collective Intergenerational Trauma	OR	Collective Transgenerational Trauma	AND	Consequences
Collective Intergenerational Trauma	OR	Collective Transgenerational Trauma	AND	Intergenerational Trauma Interventions
Intergenerational Trauma Interventions	AND	Social care	OR	Health Professionals

**Table 2:** Analysis of the attributes of the studies.

Characteristics		Studies n (%)
Asia	Era of studies	7 (31.8)
North America		5 (22.7)
Europe		3 (13.6)
Oceania		3 (13.6)
Africa		2 (9.1)
Multinational		2 (9.1)
	Year of publication	
2016–2018		5 (22.7)
2019–2021		3 (13.6)
2022–2026		14 (63.6)
	Type of studies	
Quantitative studies		11 (50.0)
Qualitative studies		8 (36.4)
Mixed-methods studies		2 (9.1)
Case study/Reflective paper		1 (4.5)

## Data Availability

No new data were created or analyzed in this study. Data sharing is not applicable to this article.

## Appendix A

**Table A1:** Research studies by Author(s), Title, Year, Country, Aim, Sample size, Study design and Finding(s).

Author	Title	Year	Country	Aim-Variables	Sample Size	Study Design	Outcomes
[25]	Intergenerational trauma transmission through family psychosocial factors in adult children of Rwandan survivors of the 1994 genocide against the Tutsi	2024	Rwanda	Familial and psychosocial mechanisms involved in the intergenerational transmission of trauma among descendants of genocide survivors.	251 adult descendants	Quantitative study	Intergenerational trauma transmission was associated with parental trauma communication, dysfunctional family communication, and negative parenting practices, while post-traumatic stress, depression, and anxiety were observed among descendants.
[26]	Examining intergenerational transmission of Holocaust trauma as it relates to Jewish identity, communication type, and mental well-being	2022	United States	The relationship between Jewish identity, family communication about the Holocaust, historical loss, and descendants’ mental well-being.	98 adult descendants	Quantitative study	Family communication transmitted trauma, while indirect and guilt-inducing communication was associated with lower mental well-being.
[27]	Living alongside past trauma: Lived experiences of Australian grandchildren of Holocaust survivors	2022	Australia	The lived experiences and mechanisms of intergenerational trauma transmission among grandchildren of Holocaust survivors.	14 grandchildren	Qualitative study	Family communication and historical memory contributed to trauma transmission, while the traumatic past continued to affect descendants’ emotional reactions and lives.
[28]	So You, My Children, Can Have a Better Life: A Cambodian American Perspective on the Phenomenology of Intergenerational Communication about Trauma	2016	United States	How young Cambodian Americans learn about their families’ traumatic experiences and the factors shaping intergenerational communication.	13 young adults	Qualitative study	Knowledge of the traumatic past was transmitted through family, community narratives, and the media, while emotional factors shaped the quality of intergenerational communication.
[29]	Intergenerational Trauma and Its Relationship to Mental Health Care: A Qualitative Inquiry	2020	Australia	Psychiatrists’ perceptions and management of intergenerational trauma in clinical practice and the factors influencing its manifestation and maintenance.	13 psychiatrists	Qualitative study	Intergenerational trauma was transmitted through parental psychological distress, reduced parenting capacity, disrupted attachment patterns, repeated trauma cycles, and broader sociocultural consequences.
[30]	Silencing or silent transmission? An exploratory study on trauma communication in Kurdish refugee families	2024	Belgium	Communication of collective violence, exile, and trauma within Kurdish refugee families, focusing on family communication, narratives, collective memory, and parent–child interactions.	10 parents and 17 children	Qualitative study	Trauma was transmitted indirectly through silence, discussions about violence, collective history, family narratives, cultural myths, and symbolic objects.
[31]	Multigenerational legacies of trauma: Modeling the what and how of transmission.	2016	Israel and North America	What is transmitted from Holocaust survivors to their children and the familial and post-traumatic mechanisms involved.	422 adult descendants	Quantitative study	Transmission was associated with parental post-traumatic adjustment and disrupted intergenerational bonds, while the sociocultural environment had a protective effect.
[32]	Perceptions of aging among middle-aged offspring of traumatized parents: The effects of parental Holocaust-related communication and secondary traumatization	2016	Israel	Parental communication about the Holocaust and secondary traumatization as mechanisms of intergenerational trauma transmission among descendants.	450 participants	Quantitative study	Intrusive parental communication was associated with increased secondary traumatization, while informational communication was not associated with negative effects.
[33]	Children of Holocaust Survivors: The Experience of Engaging with a Traumatic Family History	2023	Australia	Adult children’s experiences of and engagement with the traumatic family history of Holocaust survivors.	24 siblings	Qualitative study	Traumatic family history was transmitted through direct and indirect communication, silence, and fragmented information, causing emotional discomfort while also promoting meaning-making and the preservation of historical memory.
[34]	A Qualitative Exploration of Transgenerational Trauma and Cultural Identity of Armenian Genocide Descendants.	2017	United States	The intergenerational impact of the Armenian Genocide on the cultural identity and lived experiences of Armenian American descendants.	14 descendants	Qualitative study	Storytelling and knowledge transmission were key mechanisms of intergenerational transmission, while the traumatic legacy was associated with negative effects, resilience, cultural pride, and strengthened Armenian identity.
[36]	Intergenerational Trauma in the Context of the 1947 India–Pakistan Partition	2023	India	Intergenerational trauma and adaptation among children and grandchildren of survivors of the India–Pakistan Partition.	62 descendants	Quantitative study	Both generations showed moderate levels of intergenerational trauma, with no significant differences in trauma, parental post-traumatic adjustment, or reparative effects.
[37]	Social cognition in refugee children: An experimental cross-sectional study of emotional processing with Syrian families in Turkish communities.	2021	Turkey	The relationship between post-traumatic stress among Syrian refugee parents and their children’s emotional processing abilities.	100 families (394 participants)	Quantitative study	Maternal post-traumatic stress was associated with reduced emotion recognition among children, while no corresponding relationship was found for paternal post-traumatic stress.
[38]	Intergenerational trauma and resilience among second-generation Southeast Asian Americans	2024	United States	Experiences of intergenerational trauma, resilience, cultural identity, and collective healing among second-generation Southeast Asian American adults.	15 adults	Qualitative study	Intergenerational trauma was associated with identity difficulties, anxiety, emotional detachment, and cultural conflicts, while also strengthening bicultural identity and resilience.
[39]	Intergenerational perceptions of mass trauma’s impact on physical health and well-being	2018	Ukraine	The intergenerational consequences of famine-genocide-related mass trauma on the physical health and well-being of survivors and descendants.	45 participants	Qualitative study	Mass trauma negatively affected the physical health and well-being of survivors and descendants, with effects persisting across three generations.
[40]	Complex posttraumatic stress disorder in intergenerational trauma transmission among Eritrean asylum-seeking mother-child dyads	2024	Israel	The relationship between maternal CPTSD, PTSD, and depression and children’s internalizing and externalizing difficulties.	127 mothers	Quantitative study	Maternal CPTSD was associated with increased internalizing and externalizing difficulties, including anxiety, social withdrawal, aggression, and emotional regulation difficulties among children.
[35]	The role of maternal trauma and discipline types in emotional processing among Syrian refugee children.	2023	Turkey	The relationship of maternal post-traumatic stress and parental disciplinary practices with children’s emotional processing in Syrian refugee families.	212 children and 94 mothers	Quantitative study	Maternal psychopathology contributed to intergenerational trauma transmission, while children exhibited difficulties in emotional processing and emotion regulation.
[41]	The feasibility and preliminary efficacy of narrative exposure therapy on post-traumatic stress disorder among Syrian refugees in Jordan.	2023	Jordan	The preliminary efficacy, feasibility, and acceptability of NET in reducing PTSD, anxiety, and depression among adult Syrian refugees.	40 adults	Quantitative study	NET reduced PTSD, anxiety, and depression symptoms, with high treatment completion rates and participant satisfaction.
[42]	An initial evaluation of narrative exposure therapy as a treatment of posttraumatic stress disorder among Sudanese refugees in Cairo, delivered by lay counselors.	2022	Egypt/Cairo	The feasibility and therapeutic outcomes of NET delivered by trained lay counselors for Sudanese refugees with PTSD.	7 adults	Mixed-methods study	NET significantly reduced PTSD, anxiety, and psychological distress and was considered a feasible and acceptable intervention.
[43]	Culturally adapted cognitive behavioral therapy plus problem management (CA-CBT+) with Afghan refugees: A randomized controlled pilot study.	2020	Germany	The effectiveness of CA-CBT+ in reducing psychological distress, PTSD, depression, and somatization and improving emotional regulation and quality of life.	24 adults	Quantitative study	CA-CBT+ reduced psychological distress, PTSD, depression, and somatic symptoms and improved emotional regulation and quality of life, with benefits maintained one year after the intervention.
[44]	Intergenerational Dialogue and Inquiry for Collective Healing, Social Justice and Communal Well-Being: A Reflection on Conceptions, Processes and Practices	2025	Multinational; 9 communities across 4 continents	The contribution of Intergenerational Dialogue and Inquiry (IDI) to breaking intergenerational silence, restoring cultural memory, and enhancing collective healing and community well-being.	650 participants	Case study	IDI strengthened cultural identity and intergenerational relationships while promoting collective healing and community well-being.
[45]	Investigating Outcomes of a Family Strengthening Intervention for Resettled Somali Bantu and Bhutanese Refugees: An Explanatory Sequential Mixed Methods Study	2022	United States, New England	The effectiveness, feasibility, and acceptability of FSI-R in addressing psychological and familial consequences of collective trauma among refugee families.	80 families	Mixed-methods study	FSI-R improved family functioning and reduced children’s psychological and behavioral difficulties and was considered feasible and acceptable.
[46]	Effectiveness of the Caregiver Support Intervention on Child Psychosocial Wellbeing Among Syrian Refugees in Lebanon: Mediation and Secondary Analysis of a Randomized Controlled Trial	2025	Lebanon	The effectiveness of CSI in reducing the psychosocial and familial consequences of collective trauma.	240 caregivers	Quantitative study	CSI improved children’s and caregivers’ well-being and reduced harsh parenting behavior, but the benefits were not maintained at the three-month follow-up.
